# Land use to agriculture and planted forests strongly affect the genetic diversity of *Baccharis crispa* Spreng., a native herb of South America

**DOI:** 10.1093/aobpla/plae050

**Published:** 2024-09-13

**Authors:** Ricardo Micolino, Felipe Górski, Felipe Liss Zchonski, Rhaniel Nicholas Lisowski Gonçalves, Juliana da Rosa, Paulo Roberto Da-Silva

**Affiliations:** DNA Laboratory, Universidade Estadual do Centro-Oeste, UNICENTRO, Alameda Élio Antonio Dalla Vecchia, 838, Guarapuava, Paraná, 85040-167, Brazil; DNA Laboratory, Universidade Estadual do Centro-Oeste, UNICENTRO, Alameda Élio Antonio Dalla Vecchia, 838, Guarapuava, Paraná, 85040-167, Brazil; DNA Laboratory, Universidade Estadual do Centro-Oeste, UNICENTRO, Alameda Élio Antonio Dalla Vecchia, 838, Guarapuava, Paraná, 85040-167, Brazil; DNA Laboratory, Universidade Estadual do Centro-Oeste, UNICENTRO, Alameda Élio Antonio Dalla Vecchia, 838, Guarapuava, Paraná, 85040-167, Brazil; DNA Laboratory, Universidade Estadual do Centro-Oeste, UNICENTRO, Alameda Élio Antonio Dalla Vecchia, 838, Guarapuava, Paraná, 85040-167, Brazil; DNA Laboratory, Universidade Estadual do Centro-Oeste, UNICENTRO, Alameda Élio Antonio Dalla Vecchia, 838, Guarapuava, Paraná, 85040-167, Brazil

**Keywords:** Nature conservation, Atlantic Forest, Araucaria Forest, grasslands, natural pastures, population genetics, molecular markers, microsatellites, ISSR

## Abstract

Human population growth constantly requires an increase in the production of food and products from the timber industry. To meet this demand, agriculture and planted forests are advancing over natural areas. In view of this, it is necessary to know the effects of land use for different purposes (grain production, pastures, planted forests, fruit production and among other uses) on the genetic diversity of populations of native species. This knowledge can assist in land use planning as well as in the development of conservation strategies for native species. In this study, we evaluated the effect of land use for agriculture (mainly for cereal production) and planted forests on the genetic diversity of *Baccharis crispa* Spreng., a herb native to South America. To achieve our goals, we compared population genetic data obtained with three molecular markers (microsatellites, inter-simple sequence repeat and isoenzymes) with data on land use for agriculture and planted forests from 15 different locations. Our results showed that regardless of the molecular marker used, the greater the use of land for agriculture and planted forests, the lower was the genetic diversity of *B. crispa* populations. *Baccharis crispa* is a semi-perennial species that needs at least one year to reach its reproductive period, which is prevented in agricultural areas due to the land being turned over or dissected with herbicides every 6 months. In the studied regions, the planted forests are of eucalypt and/or pine, which besides being species with a high production of allelopathic substances, produce strong shading and *B. crispa* is a species that inhabits open grassland that needs a high incidence of sunlight for development. The data obtained in our study can assist in the decision-making to use land in order to reconcile the production of supplies for humanity and for the conservation of nature.

## Teaser

This study assessed the impact of land use for agriculture (especially cereal production) and planted forests (eucalypts and/or pines) on the genetic diversity of *Baccharis crispa* populations in South America. The results showed that the greater the use of land for agriculture and planted forests, the lower the genetic diversity of *B. crispa* populations. We also found that populations in pasture areas retain high genetic diversity. In this context, the land use for pasture can help maintain genetically viable populations of the species.

## Introduction

Changes in land use, which involve converting natural landscapes to predominantly human use, have transformed a large part of the planet’s land surface since the agricultural revolution. Land use has been identified as one of the main drivers of biodiversity change at global, national and local scales, therefore, its value is implicit in the world environment and in sustainability research (Haines-Young 2009). Landscape changes are mainly due to urbanization, pasture expansion and agricultural development (Foley *et al*. 2005). Some of the most significant negative impacts of anthropogenic changes in land use may be related to disturbances in the ecological integrity and biodiversity of natural environments. One of the most worrying factors would be the introduction of invasive species, which often also contributes to the loss of local biodiversity and includes the transition from pathogens directly to humans (Gottdenker *et al*. 2014).

Much of the diversity of an ecosystem can be extirpated by changing its properties (Pimm and Raven 2000). This may be due to the influence on reproductive and dispersal cycles of native plants, which are critical steps for maintaining populations and responding to environmental changes (Cruzan and Hendrickson 2020). In plants, land use can lead to population isolation and fragmentation, potentially resulting in genetic differentiation in subdivided populations, as well as loss of population genetic diversity (Rudmann-Maurer *et al*. 2007; Kloss *et al*. 2011). The characteristics of genetic diversity are undoubtedly recognized as fundamental to biological evolution (Ellegren and Galtier 2016). Understanding how genetic diversity in natural populations is disturbed by human activity can contribute to knowledge about the ability of a species to respond to environmental changes, in addition to facilitating the development of cultivation and conservation strategies (Rao and Hodgkin, 2002).

Anthropized landscapes in which cultivated and native species coexist tend to force many native species to interact with agricultural systems and to depend on semi-natural habitats to maintain their gene pool (Pimentel *et al*. 1992; Schmidt *et al.* 2009). In general, the grassland areas of South America, and the herbaceous species existing in it, are neglected in terms of the impact of anthropic action. This condition is an anthropocentric view of most people who live in these regions. When people observe no trees in natural open grassland areas in southern Brazil, they do not consider it an important environment and, therefore, not suitable for conservation. However, it is known to the scientific community that the transformation of any natural landscape can affect the species diversity as well as the structure and genetic diversity of populations (Manel *et al*. 2003; Cousins and Aggemyr 2008; Cousins 2009; Schmidt *et al*. 2009; Öckinger *et al*. 2012; Milberg *et al*. 2019). For example, different land uses affected the genetic pattern of *Geum urbanum* L., influencing the stochastic processes that determine gene flow and population structure (Schmidt *et al*. 2009). The continuous process of habitat change and degradation due to the intensification of agricultural land use has forced an increasing number of plant species to survive in remaining semi-natural habitats, such as roadside vegetation (Robinson and Sutherland 2002). Such environments generally contain small and relatively isolated populations, which makes them increasingly susceptible to genetic drift and inbreeding, resulting in genetic erosion (Bijlsma and Loeschcke 2012). In Brazil, the main changes in habitats occur through human interventions to transform native areas into agricultural areas. In southern Brazil, this mainly occurs for the planting of cereals (soybeans, corn and wheat) and planted forests (eucalypt and pine). In 2017, Brazilian agribusiness exceeded 81 billion US dollars, supplying products for the domestic market to more than 150 countries (EMBRAPA 2018). To guarantee the supply of food to the world and the conservation of nature in agricultural areas, it is necessary to understand the dynamics of natural populations in view of the effect of the presence of agriculture and planted forests. In this sense, an attempt to discover how land use may be affecting the genetic diversity of native species can be through the study of populations of species that co-occur with crops (Oldfield *et al*. 2019).

**Figure 1.**
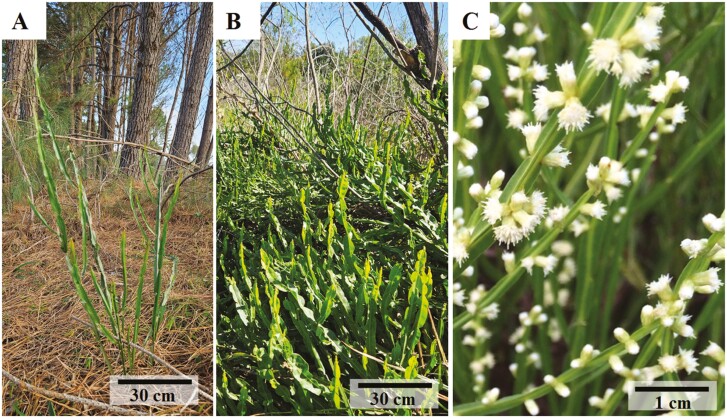
Vegetative and reproductive aspects of *Baccharis crispa* Spreng. A) Aspects of the species in a site with high percentage of land use for planted forests; B) Aspects of the species in a site without human interference (wild conditions); and C) Details of the inflorescences and the winged stem of the species.

*Baccharis crispa* Spreng. (known as Carqueja) is a herbaceous species (Fig. 1), native to South America (Barroso 1976), with cross-fertilization and seed dispersal predominantly by anemochory (Auler *et al*. 2004). Despite being herbaceous, it reaches the reproductive phase after one year of germination. In Brazil, its area of occurrence covers the Atlantic Forest, Cerrado and Pampa biomes (Rodrigues and Carvalho 2001; Vieira and Silva 2002; Ritter and Baptista 2005). Because it is a heliophile species, it prefers a variety of habitats in open areas and forest edges as well as in pastures. It is also commonly found in heavily anthropized areas, such as roadsides, abandoned land and small areas on the border of agricultural farms where the soil is not revolved annually. Thus, the species is representative of the semi-natural vegetation that occurs in agricultural landscapes in southern Brazil. *Baccharis crispa* stands out ecologically for being a pioneer and melliferous species with resistance to natural grassland environments disturbed by anthropogenic actions (Burg and Mayer 2002; Castro *et al*. 2002). Economically it stands out for the wide commercialization of its stem for making teas with medicinal properties (Castro *et al*. 2002), and is one of the most used medicinal plants in southern Brazil. The characteristics of wide distribution, long initial life cycle and occurrence in open and/or anthropized areas make this species an adequate model to examine the consequences of land use on the genetic diversity of natural populations in open grassland areas.

In this study, our main objective was to investigate the influence of land use, particularly for agriculture (cereal production) and planted forests, on the genetic diversity of *B. crispa* populations. We hypothesized that there will be a correlation between the intensity of land use and the genetic diversity of populations. Thus, we tested for the correlation, if any, between the percentage of land use with the polymorphism and genetic diversity generated from three independent molecular markers.

## Materials and Methods

### Populations evaluated with ISSR and microsatellite markers

**Figure 2.**
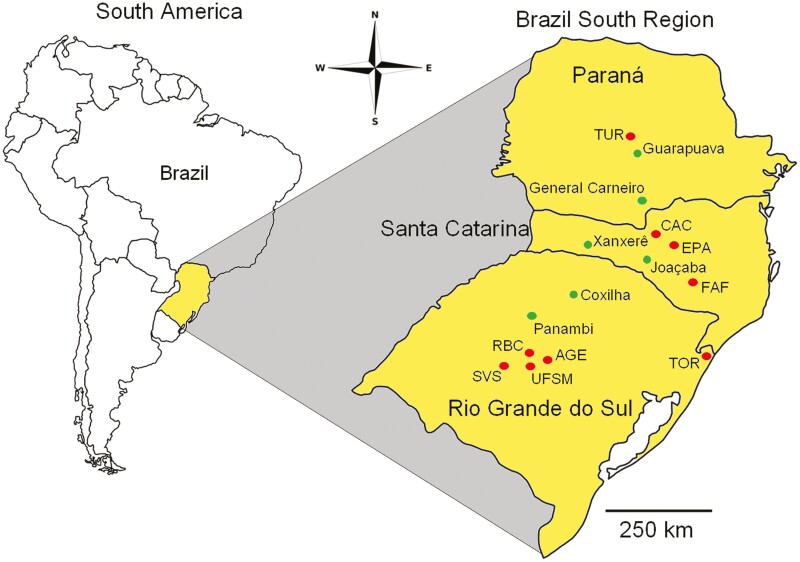
A map of South America with emphasis on the Southern region of Brazil with the sampling sites of *Baccharis crispa* Spreng. populations. In the sampling sites identified by abbreviated names in all capital letters, populations were evaluated using isoenzymatic markers. At the other sites, populations were assessed with microsatellite and ISSR markers. UFSM: Universidade Federal de Santa Maria, AGE: Arroio Grande, RBC: Reserva Biológica da CORSAN, SVS: São Vicente do Sul, TOR: Torres, FAF:| Fazenda Amola Faca, CAC: Caçador, EPA: EPAGRI Caçador, TUR: Turvo.

Six populations of *B. crispa* were collected from the southern region of Brazil for analyses with inter-simple sequence repeat (ISSR) and microsatellite markers (Fig. 2). Stems from 16 to 21 individuals, spaced at least 15 m apart, were collected from each population and packed in silica gel until DNA extraction.

Before DNA extraction, the stems were crushed in liquid nitrogen until a very fine powder was obtained. DNA extraction was performed according to the methodology proposed by Doyle and Doyle (1987). In the tubes containing 200 mg of crushed plant material, 700 μL of buffer composed of 20 mM EDTA (Ethylenediamine tetraacetic acid), 0.1 M Tris-HCl pH 8.0 (tris(hydroxymethyl)aminomethane), 1.4 M NaCl (Sodium chloride), 2% CTAB (Cetyl trimethylammonium bromide) and 0.4% b-mercaptoethanol, were added. After extraction the DNA was dried and resuspended in 100 μL of TE buffer and treated with RNAse at 10 mg mL^-1^. The DNA samples were quantified by electrophoresis on 0.9% agarose gel stained with ethidium bromide, using standard known quantities of Phage λ DNA (50; 100; 200 and 400 ng) as a comparison.

For analysis with DNA-based markers, 15 ISSR primers and six primers pairs of microsatellite were used (Table 1). Polymerase chain reaction (PCR) amplifications with ISSR markers and electrophoresis followed the protocols proposed by Rosa *et al*. (2017). Amplification reactions were conducted in a final volume of 12.5 μL that contained 20 ng DNA, 0.2 μM each primer, 200 μM dNTPs, 1.5 mM MgCl_2_, 1 U Taq DNA Polymerase, 1× PCR buffer and water to complete the volume. The program for amplification consisted of an initial denaturation step at 94 °C for 5 min, followed by 35 cycles of 90 °C for 45 s, annealing temperature of the primer (Table 1) for 45 s, and 72 °C for 60 s. After the cycles, a final step of 72 °C for 7 min was added for the complete extension of the fragments. The amplification products were separated by electrophoresis on 1.8 % agarose gel at a constant voltage of 110 V for 2 h. The gels were stained with ethidium bromide and visualized under UV light. To determine the size of the amplified fragments, a 100-bp DNA ladder was used. The microsatellite markers used in this study were initially isolated as follows: SS20E and SS24F from *Solidago sempervirens* L. (Wieczorek and Gerber 2002), EARI4-6 from *Erigeron arisolius* G.L. Nesom (Lindsay *et al*. 2012), SG_2 and SG_8 from *Solidago gigantea* Aiton (Beck *et al.* 2014) and ER-HAJZC from *Erigeron rupicola* Phil (Takayama *et al*. 2012).

**Table 1.** List of 15 ISSR and six microsatellites primers used to obtain population genetic data from *Baccharis crispa* Spreng. populations with their respective sequences and annealing temperatures (T°C).

ISSR			Microsatellite		
Primer	Sequence^*^ 5ʹ–3ʹ	T°C	Primer	Sequence 5ʹ–3ʹ	T°C
UBC807	(AG)_8_T	55	SS20E	CACACAGACACTCAAAGCTTCA	50
UBC808	(AG)_8_C	50		ACCCGCCCTAAAAATAAAGA	
UBC823	(TC)_8_C	52	SS24F	AGCTTTTCTTCGCCATTTCCTTCC	59
UBC824	(TC)_8_G	50		AATTTGGTTACTGGGTTTTCTTGA	
UBC827	(AC)_8_G	50	EARI4-6	GCGGTTTGTGTAGAAGTCC	57
UBC834	(AG)_8_YT	52		ATCTCACTGGTGAATTTCAGAG	
UBC843	(CT)_8_RA	54	SG_2	TCTAAACTGTAAGTCTTTGATGAAAC	55^**^
UBC848	(CA)_8_RG	55		GCCGTCAATCCTTACAATCC	
UBC855	(AC)_8_YT	50	SG_8	TCCCTCTTTATTCTTTCAACAAACC	55^**^
UBC856	(AC)_8_YA	55		GTTT AACACCAACATTGCAATCCC	
UBC859	(TG)_8_RC	55	ER-HAJZC	GGATATCGGTTTGGCTTGA	55^**^
UBC866	C(TCC)_5_TC	55		GGAATCCCTTCTCTTTCTGA	
UBC868	(GGA)_6_	53			
UBC873	(GACA)_4_	50			
UBC899	CATGGTGTTGGTCATTGTTCCA	55			

^*^Y = (C,T); R = (A, G),

^**^Touchdown cycles.

Microsatellite markers amplification by PCR and electrophoresis followed the protocols proposed by Weber *et al*. (2020). The amplification was performed in a final volume of 10 μL containing 1× PCR buffer, 3 mM MgCl_2_, 0.2 mM dNTP, 0.8 mM each of forward and reverse primers, 0.4 U Taq DNA Polymerase, 20 ng DNA and ultrapure water (to complete the volume). The DNA amplification by PCR was carried out in a thermal cycler programmed for initial denaturation at 96 °C for 3 min, followed by 35 cycles of 94 °C for 30 s, primer annealing temperature (Table 1) for 1 min, and 72°C for 1 min, and a final extension at 72 °C for 15 min. For primer pairs SG_2, SG_8 and ER-HAJZC, a touchdown program was used starting at 60 °C, decreasing 1 °C per cycle until reaching 55 °C. The amplified PCR products were separated by electrophoresis in a 3 % agarose gel at a constant 110 V current for 4 h and visualized under UV light by staining with ethidium bromide (0.5 μg/mL^−1^). To determine the size of the amplified fragments, a DNA ladder 100–bp marker was used. The agarose used (Sigma-Aldrich, catalog number A4718) is ideal for the separation of small DNA fragments differing in size by as little as 2 % and compares to the resolution of DNA in polyacrylamide gels.

The gels obtained after electrophoresis were visually evaluated and genotypic matrices were produced with the data for each marker. From the matrices, for each population, using POPGENE software version 1.31 (Yeh *et al*. 1999), percentage of polymorphisms, genetic diversity of Nei (*h*), and gene flow between populations were calculated with the data obtained with ISSR, and percentage of polymorphisms, expected heterozygosity (*He*) and gene flow between populations were calculated with data obtained with microsatellites markers. The genotypic matrices obtained with the ISSR and microsatellites markers of each population are available in Supplementary Information—S1.

### Populations evaluated with isoenzymatic markers

For the studies with isoenzymatic markers, the data of the expected heterozygosity, polymorphism and geographic location of each population were extracted from Auler (2004). This author evaluated 10 enzyme loci in 300 individuals from 10 populations (30 individuals per population). Genotyping was performed on 13 % penetrose gel. In our analysis, we did not include the Turvo manejada (TUM) population referenced by Auler (2004) because it is not a natural population.

### Survey of land use

**Figure 3.**
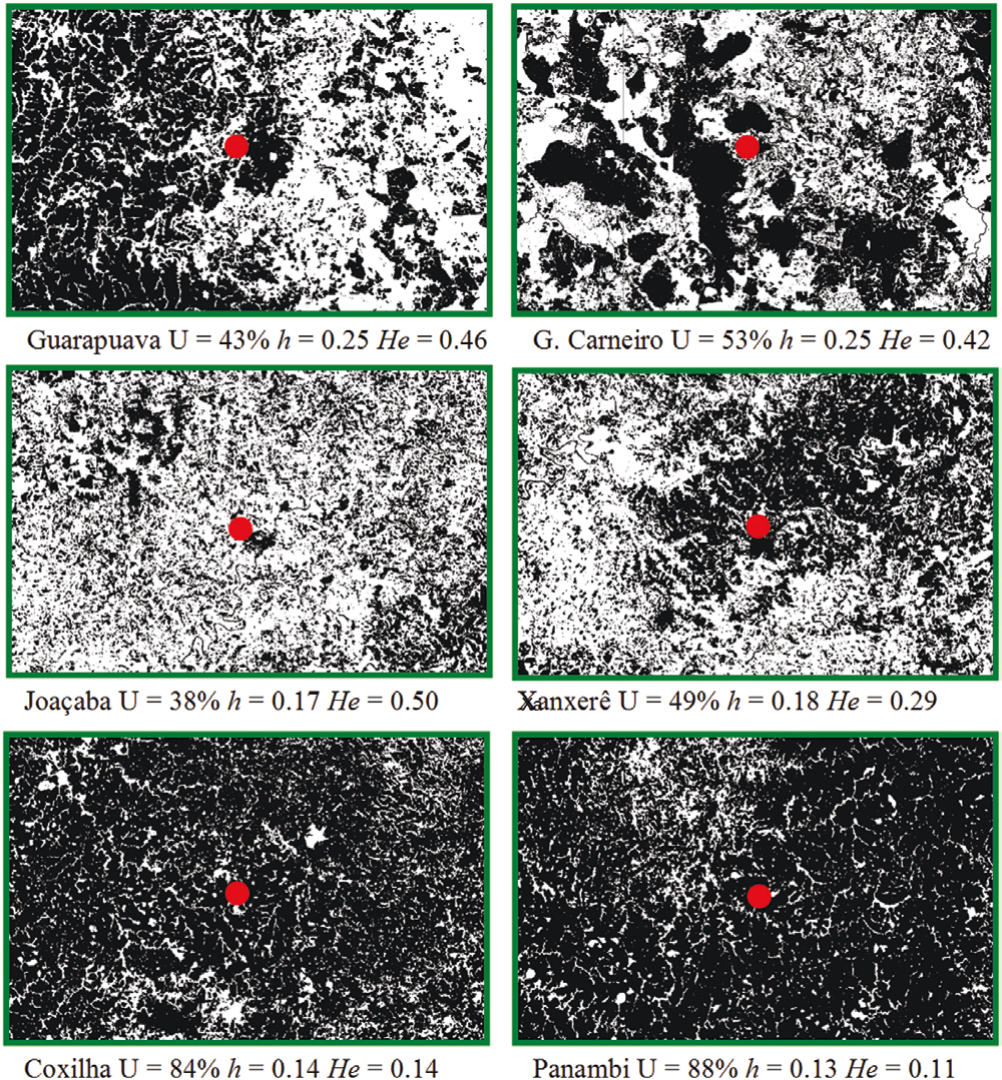
Sampling sites and land use for agriculture and planted forests (black areas in the green squares) in the regions from where *Baccharis crispa* Spreng populations were collected. The red dot indicates the sampling site for each population. The green outlined area of each square represents 2146 km^2^. Land use data were obtained from the MapBiomas platform (http://mapbiomas.org/). U: percentage of land use for agriculture and planted forests, *h*: Nei genetic diversity, and *He*: expected heterozygosity.

To survey the land use data at the sampling site of each population, a MapBiomas platform (Brazilian Annual Land Use and Land Cover Mapping Project) available at http://mapbiomas.org/, was used. This platform presents land use data with a resolution of 1 metre. After accessing the geographical coordinates of each population on the platform, the layers of agriculture and planted forests were selected. With the selected layers, an area of 2146 km^2^ was extracted around the sampling site of each population (Fig. 3). The image obtained was converted to 8-bit resolution with a black/white ratio of 0/217 217 using the ImageJ 1.51j8 (National Institutes of Health, Bethesda, MD, USA) software. Following this, using the same software, the percentage of pixels in the figure was calculated, which corresponds to the area occupied by agriculture and planted forests.

### Statistical analysis

The data obtained from polymorphism (P), and Nei genetic diversity (*h*) (from ISSR markers) or expected heterozygosity (*He*) (from microsatellite and isoenzymatic markers) of each population and for each marker was tested for correlation with the percentage of land use for agriculture and planted forests using Pearson’s coefficient (*r*). Linear data regression was also performed and the determination coefficient (*R*^*2*^) was calculated. The correlation between gene flow of the pairs of populations and geographical distance and the *R*^*2*^ coefficient of this correlation were obtained by the Mantel test using the NTSYS-pc software (Rohlf 2000). The analyses related to gene flow were not performed with the populations evaluated by Auler (2004) because this information was not available.

## Results

The 15 ISSR primers used in the six populations of *B. crispa* amplified 195 loci, with an average of 13 loci per primer, and of these, 172 were polymorphic and used to calculate the percentage of polymorphism and genetic diversity. The populations of General Carneiro, Coxilha and Panambi were those with the lowest genetic diversity and polymorphism (Table 2). The six microsatellite primers pairs used amplified 22 alleles with an average of 3.66 alleles per locus. As observed for ISSR markers, with the data from microsatellite markers, the populations of Coxilha and Panambi showed the lowest values of genetic diversity, but the Xanxerê population joined these populations with the lowest genetic indices, instead of the population of General Carneiro (Table 2). The percentage of land use for agriculture and planted forests was higher in the sampling sites of the populations of Panambi, Coxilha and General Carneiro (Table 2; Fig. 3).

**Table 2.** Polymorphism values, Nei genetic diversity (*h*) and expected heterozygosity (*He*) in natural populations of *Baccharis crispa* Spreng. and percentage of land use for agriculture and planted forests in the regions where the populations were collected. N: sample size; % P: percentage of polymorphism.

Population	*N*	ISSR		Microsatellite		Use	Geographic coordinate
		h	%P	He	%P	%	
Guarapuava	16	0.25	77	0.46	83	43	25°25ʹ03.44ʹS 51°30ʹ30.32ʹW
General Carneiro	16	0.13	44	0.42	83	53	26°26ʹ55.47ʹS 51°22ʹ36.82ʹW
Joaçaba	19	0.17	59	0.50	100	38	27°07ʹ40.57ʹS 51°36ʹ58.58ʹW
Xanxerê	20	0.18	55	0.29	67	49	26°51ʹ03.76ʹS 52°31ʹ38.54ʹW
Coxilha	21	0.14	45	0.14	50	84	28°05ʹ32.57ʹS 52°16ʹ34.12ʹW
Panambi	20	0.13	48	0.11	67	88	28°22ʹ43.54ʹS 53°21ʹ44.96ʹW

Gene flow was variable between populations, with higher values obtained with ISSR markers (Table 3). With both markers, the populations of Coxilha and Panambi showed the lowest values of gene flow (Table 3). Pearson’s correlation between gene flow obtained with ISSR markers and geographical distance was positive (*r* = 0.03) and *R*^*2*^ = 0.00. For the gene flow obtained with the microsatellite markers and the geographical distance, the correlation was negative (*r* = − 0.50) and the *R*^*2*^ = 0.25.

**Table 3.** Gene flow (above the diagonal) and geographic distance in kilometres (below the diagonal) between the six populations of *Baccharis crispa* Spreng. evaluated in this study using ISSR and microsatellite markers. Above the diagonal the numbers before the slash (/) are those obtained with the ISSR markers and after the slash are those obtained with the microsatellites.

	Guarapuava	G. Carneiro	Joaçaba	Xanxerê	Coxilha	Panambi
Guarapuava		3.54/0.64	3.62/0.75	3.72/0.72	2.41/0.22	2.46/0.30
General Carneiro	112		2.3/2.02	2.2/0.42	1.33/0.39	1.48/0.45
Joaçaba	188	82		2.02/1.74	1.16/0.53	1.32/0.63
Xanxerê	187	123	95		1.32/0.36	1.41/0.53
Coxilha	305	205	125	140		1.23/4.05
Panambi	376	292	220	190	110	

In the data obtained by Auler (2004), the values of expected heterozygosity (*He*) were lower in the populations of Turvo, Torres and Caçador (Table 4). Furthermore, the sampling regions of these populations were those with the highest land use for agriculture and planted forests (Table 4).

**Table 4.** Values of polymorphism and expected heterozygosity (*He*) obtained with the use of isoenzymatic markers in natural populations of *Baccharis crispa* Spreng. and percentage of land use for agriculture and planted forests in the regions from where the populations were sampled. N: sample size; %P: percentage of polymorphism; %U: percentage of land use. The N, *He*, %P and Geographic coordinate data was extracted from Auler (2004). The %U was obtained in our study.

Population	N	*He*	%P	%U	Geographic coordinate
Universidade Federal de Santa Maria	30	0.26	80	38	29°43ʹ08.57ʹS 53°43ʹ34.76ʹW
Arroio Grande	30	0.25	70	43	29°39ʹ30.56ʹS 53°39ʹ10.66ʹW
Reserva Biológica da CORSAN	30	0.22	80	40	29°31ʹ18.11ʹS 53°45ʹ43.47ʹW
São Vicente do Sul	30	0.30	90	38	29°42ʹ14.81ʹS 54°43ʹ07.06ʹW
Torres	30	0.18	60	59	29°20ʹ22.74ʹS 49°45ʹ19.43ʹW
Fazenda Amola Faca	30	0.25	80	40	27°46ʹ52.81ʹS 50°28ʹ13.23ʹW
Caçador	30	0.19	50	56	26°44ʹ07.89ʹS 51°11ʹ09.13ʹW
EPAGRI Caçador	30	0.23	70	43	26°44ʹ15.59ʹS 50°59ʹ51.05ʹW
Turvo	30	0.16	60	47	25°03ʹ17.04ʹS 51°31ʹ31.35ʹW

**Figure 4.**
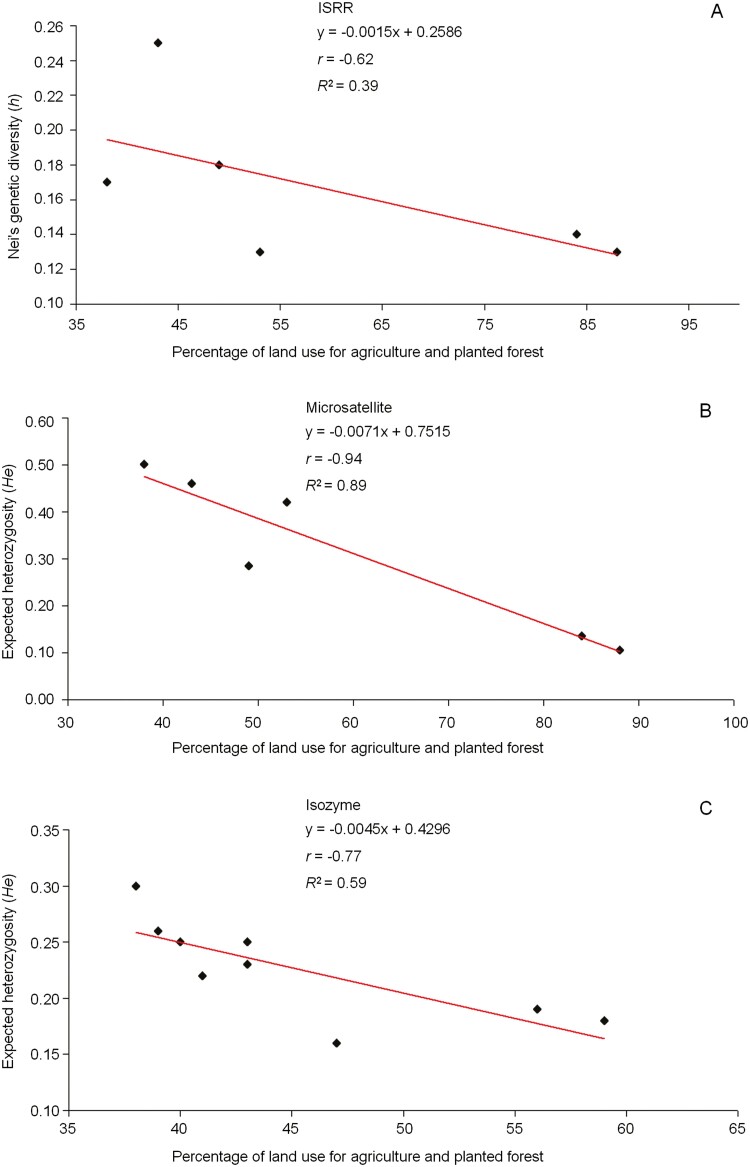
Linear regression, Pearson correlation (*r*) and coefficient of determination (*R*^*2*^) between the genetic diversity of *Baccharis crispa* Spreng. populations and the percentage of land use for agriculture and planted forests. A) Genetic diversity data obtained with ISSR markers; B) Expected heterozygosity data obtained with microsatellite markers; and C) Expected heterozygosity data obtained with isoenzyme markers.

Pearson’s correlation (*r*) was negative between genetic diversity (*h*, *He*) obtained with the three markers and the percentage of land use for agriculture and planted forests (Fig. 4). The values of *r* and *R*^*2*^ for the percentage of polymorphism obtained with the ISSR markers were the same as those obtained for *h* (*r* = − 0.62 and *R*^*2*^ = 0.39). For microsatellite markers, the values were *r* = − 0.89 and *R*^*2*^ = 0.79, and for isoenzymes, the values were *r* = − 0.88 and *R*^*2*^ = 0.77.

## Discussion

Understanding the effects of human-modified landscapes on the genetic diversity of native species is crucial for designing strategies that balance economic development with nature conservation. Numerous studies have shown that human-modified landscapes, which lead to the isolation of natural populations, negatively impact both species diversity and genetic diversity. (Vellend 2004; Vellend *et al*. 2007; Helm *et al*. 2009; Struebig *et al*. 2011; Almeida-Rocha *et al*. 2020; Naaf *et al*. 2021). Among the different types of human-modified landscape, pastures, when compared with agriculture and intensive timber production, appear to be the least harmful to natural populations, especially for pioneer and native grassland species (Naaf *et al*. 2022; Silveira-Santos *et al*. 2022). In this context, we evaluated the effect of three types of human-modified landscape (agriculture, planted forest and pastures) on the genetic diversity of *B. crispa*, a herb native from South America grassland.

Our data showed that with a higher percentage of area occupied by agriculture and planted forest, the genetic diversity of the populations of *B. crispa* was lower. It is known to the scientific community that the use of land for agricultural activities generates disturbances that can homogenize the surrounding plant communities in several ways (Foster *et al*. 1998). For instance, changes can occur in microclimates, and in soil properties, leading in turn to a variation in species composition (beta diversity) (Foster *et al*. 1998). Such an association has been observed mainly for forest species directly impacted by intensive primary agricultural activity (deforestation). In addition, constant disturbances caused due to human activity can restrict the process of recolonization of newly arrived species in the area (Vellend 2004; Vellend *et al*. 2007), and also isolate populations. These events can lead to a decrease in gene flow, thereby increasing inbreeding, and consequently, leading to a decrease in genetic diversity. These events are probably limiting the maintenance of genetic diversity in the populations of *B. crispa* present in anthropized areas with high land use for agriculture and/or forests evaluated by us.

The gene flow among the populations of *B. crispa* from Coxilha and Panambi with the other populations were the smallest regardless of the markers used (ISSR or microsatellite). Life history characteristics, especially those that affect gene flow through seeds and pollen dispersion, can affect population genetic variation (Hamrick and Godt, 1997). It is important to note that there was no correlation between geographical distance and gene flow in the studied populations of *B. crispa*. Even with the data from microsatellite markers with *r* = − 0.50, *R*^*2*^ showed that the applied model explains only 25% of the result, that is, geographic distance is not the main factor that explains the gene flow values. The populations of *B. crispa* from Coxilha and Panambi evaluated here are in highly agricultural areas, with land use for this purpose above 80 % (Fig. 3). This condition results in small populations of the species, as they only survive in places where they are able to reach the reproductive phase in small areas on the side of roads, waterlogged areas and borders of farms, where the soil is not revolved annually. Small and isolated populations are known to be inefficient in dispersion and this may be influencing the gene flow of these populations with the others. This condition of isolation, and consequent limited gene flow, may be a decisive factor in the low genetic diversity observed in these populations, and probably enhances inbreeding.

In fact, the populations of *B. crispa* found under strong pressure from agriculture, namely Coxilha and Panambi (> 80 % of land use), had the lowest rates of genetic diversity in both markers (Table 2). This result can also be strongly corroborated when observing the data with isoenzymatic markers for other populations (Table 3). The strong agricultural pressure in the southern region of Brazil is not by chance, as it represents only 6.80 % of the national territory and corresponds to 28.67 % of the temporary agricultural areas in Brazil (IBGE 2020). In the State of Rio Grande do Sul, from where such populations of less genetic diversity were collected, within the period from 2000 to 2018, 58 % of the open grassland vegetation areas were converted into agricultural areas, in addition to 18.8 % in the planted forest area (IBGE 2020). Consequently, the progressive introduction and expansion of monocultures in this region has led to a mischaracterization of natural landscapes and, contrary to what was expected, losses of Gross Domestic Product have been reported in the region, mainly due to the agricultural sector (IBGE 2020). Such conditions of absolute disturbance in land use impose a high pressure on the native herbaceous species that occupy the grassland areas of southern Brazil. Further, the use of land for agriculture, mainly cereal production, is even more drastic for species that need periods longer than six months to reach the reproductive phase, such as *B. crispa*. Thus, disturbances from agricultural areas, such as recurrent soil plowing and desiccation of vegetation by herbicides, tend to prevent the development of *B. crispa* individuals until the reproductive phase, which may be contributing to the low genetic diversity of populations from agricultural areas.

The fact that *B. crispa* is considered a pioneer species gives its populations greater persistence in habitats disturbed by human activity and yet it is suffering loss of genetic diversity. Considering this observation, the question arises as to how other species, more sensitive than *B. crispa*, are behaving in relation to these increasingly human disturbances. It is noteworthy that in agricultural landscapes, marginal populations of herbaceous, semi-shrub and shrub species are important in maintaining local diversity (Van Rossum *et al*. 2004). In this sense, the effects of agricultural areas on the pioneer species of the surrounding matrix should not be overlooked in determining the population viability of other species (Jacquemyn *et al*. 2003). *Baccharis crispa* plays an important role in maintaining pollinators for other species in its ecosystem (Barth 1990), and small populations of these species, as observed in agricultural areas, can compromise the pollinator population and, consequently, other plant species.

The population of General Carneiro showed low Nei genetic diversity (Table 2). In the region from where this population was sampled there are several planted pine and eucalypt forests. Notably, a relevant expansion of planted forest is underway, mainly in the biomes of the Southeast and South of Brazil, in the Atlantic Forest and Pampa biomes, respectively. The areas planted with eucalypts corresponded to 76 % of the forests planted for commercial purposes in Brazil, with 42 % of them concentrated in the Southeast Region, while in 2018, the largest share in the value of the national production of forestry (32.5 %) was concentrated in the South Region (IBGE 2020). It has long been known that the allelopathic properties of eucalypts and pines plantations have negative effects for a number of species around, many of them economically and ecologically important (Sasikumar *et al.* 2001; Kato-Noguchi *et al*. 2009). In the sampling region of the populations studied in this study, areas of open grassland and Araucaria Forest predominate. Both environments allow the occurrence of *B. crispa*, since the Araucaria Forest is formed by a mosaic of forest and grassland areas. Thus, the implantation of planted forests may also be interfering in the genetic diversity of populations of *B. crispa*, especially the population of General Carneiro.

*Baccharis crispa* is a species considered to be invasive in pastures (Burg and Mayer 2002; Castro *et al*. 2002). The success of the species in these areas is due to the fact that the soil is not plowed annually. This condition allows individuals of the species to reach their reproductive age and leave offspring. Due to this behaviour in pastures, the species has so far been neglected in terms of the effect of land use on species diversity. However, in our study, we observed that in areas of high land use for agriculture and/or planted forests the species is at risk, and may also compromise other species dependent on it. In this sense, efforts are needed to create areas for the conservation of the species in regions of agriculture and planted forests. In the current economic and political scenario in Brazil, it is very difficult to convince farmers to allocate part of the agricultural or planted forest areas for conservation, as this would decrease the farmer’s income. However, for some species, natural pasture areas have no negative effect on population size and genetic diversity (Kloss *et al*. 2011; Rico and Wagner *2016*; Silveira-Santos *et al*. 2022), which has also been observed for *B. crispa*. Regarding this, an alternative to help in *B. crispa* conservation is to encourage farmers to maintain natural pasture areas on farms. These areas, in addition to continuing to generate income for the farmer, will allow the conservation of *B. crispa* populations by allowing the maintenance of large populations of the species.

The major impact of human-modified areas on biodiversity is the isolation of populations into fragments, which hinders or reduces gene flow, increases homozygosity and decreases population resilience (Newbold *et al*. 2015; Miraldo *et al*. 2016; Schlaepfer *et al*. 2018). One strategy to mitigate the loss of genetic variability in populations residing in fragments is the implementation and maintenance of ecological corridors (Tambosi *et al*. 2014). Data from our study indicate that areas where the species *B. crispa* can maintain populations that complete their life cycle (which we can consider ecological corridors for the species) exhibit greater genetic diversity. This positive effect of pastures on maintaining genetic variability in native populations has been observed in other subtropical and tropical plant species (Almeida-Rocha *et al*. 2020; Silveira-Santos *et al*. 2022). In this context, encouraging the maintenance of pasture areas, forming mosaics with agricultural and planted forest areas, may help in preserving the genetic variability of native species with life cycles similar to that of *B. crispa*.

## Conclusions

The gene flow and genetic diversity of *B. crispa* is strongly and negatively influenced by the use of the land for agriculture and planted forests. This condition can compromise the survival of the species in land areas highly used for these purposes. To mitigate this condition and reduce the risk of genetic erosion in the species, the maintenance of natural pasture areas together with agricultural and planted forests areas is a promising alternative to be considered to the conservation of the *B. crispa*. A similar pattern of negative impact on genetic diversity from human-altered habitat has been observed in other species with life cycles similar to that of *B. crispa*. For example, species with specialized habitat requirements often experience reduced genetic diversity when their habitats are fragmented or altered (Aguilar *et al*. 2006; Miraldo *et al*. 2016; Forgiarini *et al*. 2024). In these cases, strategies that integrate conservation with land use, such as maintaining natural habitat corridors or implementing mixed-use landscapes that include native vegetation alongside agricultural or forestry areas, can help preserve genetic diversity. By applying such conservation strategies, not only for *B. crispa* but also for other species with similar ecological requirements, we can mitigate the adverse effects of human land use and support the long-term survival and resilience of these species in changing environments.

## Supporting Information

The following additional information is available in the online version of this article –

**Table S1.** Genotypic matrix of *Baccharis crispa* populations obtained using ISSR and microsatellite markers.

plae050_suppl_Supplementary_Files_S1

plae050_suppl_Supplementary_Table_S1

## Data Availability

All data supporting the findings of this study are available in the main text and the supporting information.
