# Supplementary figures and images for: Land use to agriculture and planted forests strongly affect the genetic diversity of Baccharis crispa Spreng., a native herb of South America
Source: AoB Plants. 2024 Sep 13;16(5):plae050. doi: 10.1093/aobpla/plae050 (PMC11445655; doi:10.1093/aobpla/plae050)

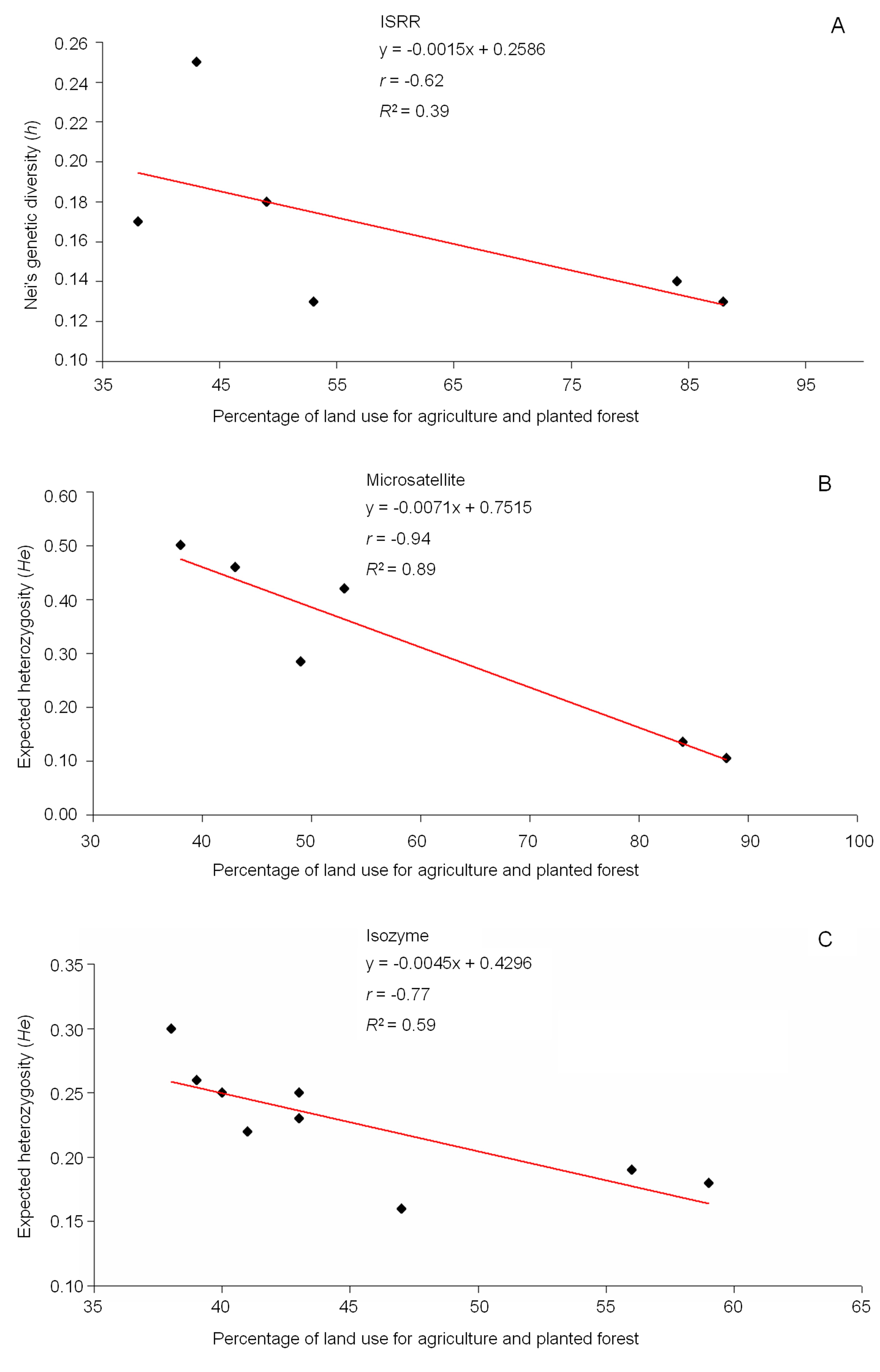

Supplement: plae050_suppl_Supplementary_Table_S1 [file plae050_suppl_supplementary_table_s1.jpeg]
